# Wild Chimpanzees on the Edge: Nocturnal Activities in Croplands

**DOI:** 10.1371/journal.pone.0109925

**Published:** 2014-10-22

**Authors:** Sabrina Krief, Marie Cibot, Sarah Bortolamiol, Andrew Seguya, Jean-Michel Krief, Shelly Masi

**Affiliations:** 1 UMR 7206 CNRS/MNHN/P7, Eco-anthropologie et d’ethnobiologie, Hommes, Natures, Sociétés, Museum national d’histoire naturelle, Paris, France; 2 Projet pour la conservation des grands singes, Kibale National Park, Fort Portal, Uganda; 3 UMR 7179 CNRS/MNHN, Mécanismes adaptatifs: des organismes aux communautés, Ecologie et de gestion de la biodiversité, Muséum national d’histoire naturelle, Paris, France; 4 UMR 7533, Dynamiques Sociales et Recomposition des Espaces, Paris Diderot University, Paris, France; 5 Uganda Wildlife Authority, Kampala, Uganda; University of Florence, Italy

## Abstract

In a rapidly changing landscape highly impacted by anthropogenic activities, the great apes are facing new challenges to coexist with humans. For chimpanzee communities inhabiting encroached territories, not bordered by rival conspecifics but by human agricultural fields, such boundaries are risky areas. To investigate the hypothesis that they use specific strategies for incursions out of the forest into maize fields to prevent the risk of detection by humans guarding their field, we carried out video recordings of chimpanzees at the edge of the forest bordered by a maize plantation in Kibale National Park, Uganda. Contrary to our expectations, large parties are engaged in crop-raids, including vulnerable individuals such as females with clinging infants. More surprisingly chimpanzees were crop-raiding during the night. They also stayed longer in the maize field and presented few signs of vigilance and anxiety during these nocturnal crop-raids. While nocturnal activities of chimpanzees have been reported during full moon periods, this is the first record of frequent and repeated nocturnal activities after twilight, in darkness. Habitat destruction may have promoted behavioural adjustments such as nocturnal exploitation of open croplands.

## Introduction

Compared to previous centuries, the level of demographic pressure and the rate of habitat loss for wildlife caused by humans have dramatically increased [1]. Today anthropogenic activities, including commercial logging, poaching, mining, illicit trade of wild animals and agricultural land encroachment are severely threatening the tropical forests and the survival of fauna, including great apes, our closest relatives. All great ape species are currently endangered and have experienced a considerable decline in population size and range in the recent years [1], [2], [3], [4]. Being charismatic umbrella species, they are widely claimed to be instrumental in the conservation of tropical forests and wildlife [5]. This emphasizes the importance of understanding and monitoring how they react and potentially adapt to different habitat changes.

While humans have been present in primate habitats since the millennia, the current rate of forest destruction and fragmentation, is resulting today in prevalent human–wildlife conflicts along protected area boundaries [6]. This situation is deteriorating further given average human population growth rates, reaching nearly double the average of rural growth at the border of some protected areas [7]. The incursions in human cultivations by forest mammals, such as elephants and primates, are therefore one of the most common behavioural responses to both habitat loss and access to new energy-rich food resources (Africa: [8], [9]; Asia: [10], [11]).

Among Primates, chimpanzees are known to be sensitive to logging due to their territoriality, and their frugivorous diet [12], [13] that leads to a lower flexibility to seasonal fluctuations in fruit availability [14]. However, chimpanzees have high cognitive abilities. They enable them to use botanical skills to discover and exploit fruits in the forest habitat according to dynamical temporal patterns [15], to access hidden food resources using tools [16], [17], [18], to cooperate to achieve a common goal (e.g. hunting, patrolling: [19], [20]) or even to use the pharmacological properties of plants to self-medicate [21], [22], [23], [24]. Nevertheless, the acquisition and transmission of such techniques and behaviours require a long period of social learning [18], [25], [26]. This long time of social learning might not fit in the rapid changes of environment and of the local population perception towards chimpanzees which occur in areas where habitat encroachment within chimpanzee habitat increases encounters with humans and consequentially chimpanzee aggressive behaviour [27]. In Western Uganda, in Bulindi, an unprotected human-dominated area, several people claimed that chimpanzees had been deliberately killed to deter crop raiding [28]. Whether chimpanzees are flexible enough to adapt their behavioural ecology and ranging patterns to their rapidly changing habitat and to the changes in the attitudes of local people regarding crop loss is a major concern for their survival. In such a fragmented farm–forest mosaic (Bulindi), a marked mobility of chimpanzees between the main forest blocks and thus crossing anthropogenic habitat is necessary even to feed on wild food [28]. In areas where chimpanzee communities inhabit a continuous forested territory that is bordered by crop fields actively guarded by humans, such as in Kibale National Park, western Uganda, first surveys suggest that the boundaries of their territory are perceived to be risky areas [29], [30]. Territoriality in chimpanzee involves the active defence of their home-range, mates and food resources from the neighbouring communities during cooperative patrols, that can sometimes be lethal [16], [31]. In Kibale National Park, chimpanzees are usually considered by local people and primatologists as infrequent crop-raiders [29], [32]. This may be either because they do really avoid such dangerous situations or because their behaviour might be so efficient as to avoid detection by humans, thus resulting in underestimation of the events [8], [29], [33]. At Bossou, chimpanzees seem to perceive the risk of human confrontation in a similar way as risk of predation consequently adapting their behavior and feeding strategies accordingly. For example, adult males who usually take more risk than females were more likely to crop-raid than females [34]. When feeding on raided food rather than wild food, chimpanzees vocalized less and showed higher frequency of signs of anxiety, including rough self-scratching, in presence of local people [34]. They also tended to transport crops into the forest to reduce stationing in exposed areas [34]. In this small community (12–14 chimpanzees), the party size was not significantly different when feeding crops or wild food but cohesiveness increased during crop-raiding [35].

In Kibale National Park, chimpanzees may also be efficient to minimize the risk of detection by farmers explaining why there are considered as infrequent. For instance, in some places, farmers report that sometimes chimpanzees visit crop fields at full moon to hide their raid ([36]; Sebitoli Chimpanzee Project, unpublished data). Chimpanzees, as other ape species are considered as strictly diurnal (illumination intensity range for feeding activity: 1–85 lux) while the illuminance (the measure of the incident light illuminating a surface), during full moon night amounts to about 0.3 lux [37], [38]. Among primates, only a few lemur and monkey species show cathemerality, thus being active during both the day and night [39], [40], [41]. To date, descriptions of night activities in wild great apes have been rare. They include night feeding activities on the night of full moon at Gombe [16], mating behaviour [42], and travelling on moonlit nights at Fongoli, Senegal [43] and Mahale, Tanzania [44]. Such behaviour has also been observed in different western gorilla groups in the attempt to avoid or escape from attacks of another group or lone male (Masi, pers. observ.).

To test the hypothesis that chimpanzees may have developed several strategies to survive in highly disturbed habitats and to avoid detection by humans, including being active during moonlit nights, we focused our survey on a maize field bordering the forested area of Sebitoli in the northern part of Kibale National Park (KNP), Uganda. In a recent review of 33 bibliographic sources, KNP is classified as “highly exposed site to agriculture” among the 27 locations scored and maize as a “cause of high conflict” out of the 51 crops eaten by chimpanzees [45]. Most of the Sebitoli chimpanzee territory (32 out of 39 km of the home range borders) is surrounded by anthropogenic landscape [46]. In addition, their home range is crossed by a tarmac road and experiences a great pressure from poaching: 40% of identified chimpanzees have limb mutilations most likely due to snares [47]. We used video-trapping method to record their behaviour in a maize field neighbouring the forested area of their territory. This non-invasive method can be used day and night with infra-red light and offers an alternative to traditional field methods based on direct observation. This method offers systematic recording of behaviours (1) in locations where chimpanzees may be reluctant to be followed by researchers due to a fear of meeting farmers, and (2) during periods where luminosity is too low to enable direct observations of chimpanzees in the case of unusual activity during full moon nights [36].

Since crop-raiding is a risky way to access valuable food resources and behavioural adaptations have been shown elsewhere [34]–[35], we predict that during crop-raiding chimpanzees would use characteristic behavioural strategies in order to avoid detection, particularly:

Hypothesis 1- chimpanzees will show specific group behaviours in response to a risky situation i.e. a) low rate of incursions, party composition and progression order into the field biased on adult males who are more often involved in risky activities, b) small party size, lower number of individuals may decrease the detectability in that large community counting about 80 members (the alternative hypothesis being that larger and more cohesive party will increase the vigilance [35])
Hypothesis 2- chimpanzees will show specific individual behaviours to limit the risk of being detected i.e. cautiousness, vigilance, being silent and extension of activity during nocturnal periods.

## Materials and Methods

### 1. Study site

Kibale National Park (795 km^2^) is a medium-altitude moist tropical forest located in Western Uganda. The landscape is a mosaic of evergreen forest, swamps, regenerating forests from former exotic softwood plantations within the national park (NP). The park is surrounded on the outside by smallholder farms, forest fragments and tea estates [48]–[49]. As a result of land scarcity and the increasing population (up to 335 ind/km^2^; [50]), agriculturalists (Batooro and Bakiga ethnic groups) have been forced to set up their lands at the edge of the national park.

### 2. Study subjects

Since 2009, the Sebitoli Chimpanzee Project monitors daily the extreme north community of chimpanzees (*Pan troglodytes schweinfurthii*) of the NP. We adhered to the research protocols defined by the Administration of Kibale National Park and all research was approved by the Museum national d’histoire naturelle. Data of this study refers to the period from the 5^th^ to 24^th^ February 2013, where a camera trap was placed at the border of their home range during the period of maize crop maturity. At this date, 72 chimpanzees were individually recognised out of an estimated number of 80 individuals. Twenty-six of them present hands, feet or limb deformities or scars resulting from being trapped in poaching snares. Eight are missing entire segment(s) of limb(s). Since 2009 *ad libitum* daily observations records and GPS waypoints of contacts with chimpanzees were used to determine the home range of the Sebitoli community by the method of Minimum Convex Polygon [51], estimated to be 25 km^2^.

### 3. Video-trap recording

**Figure 1:**
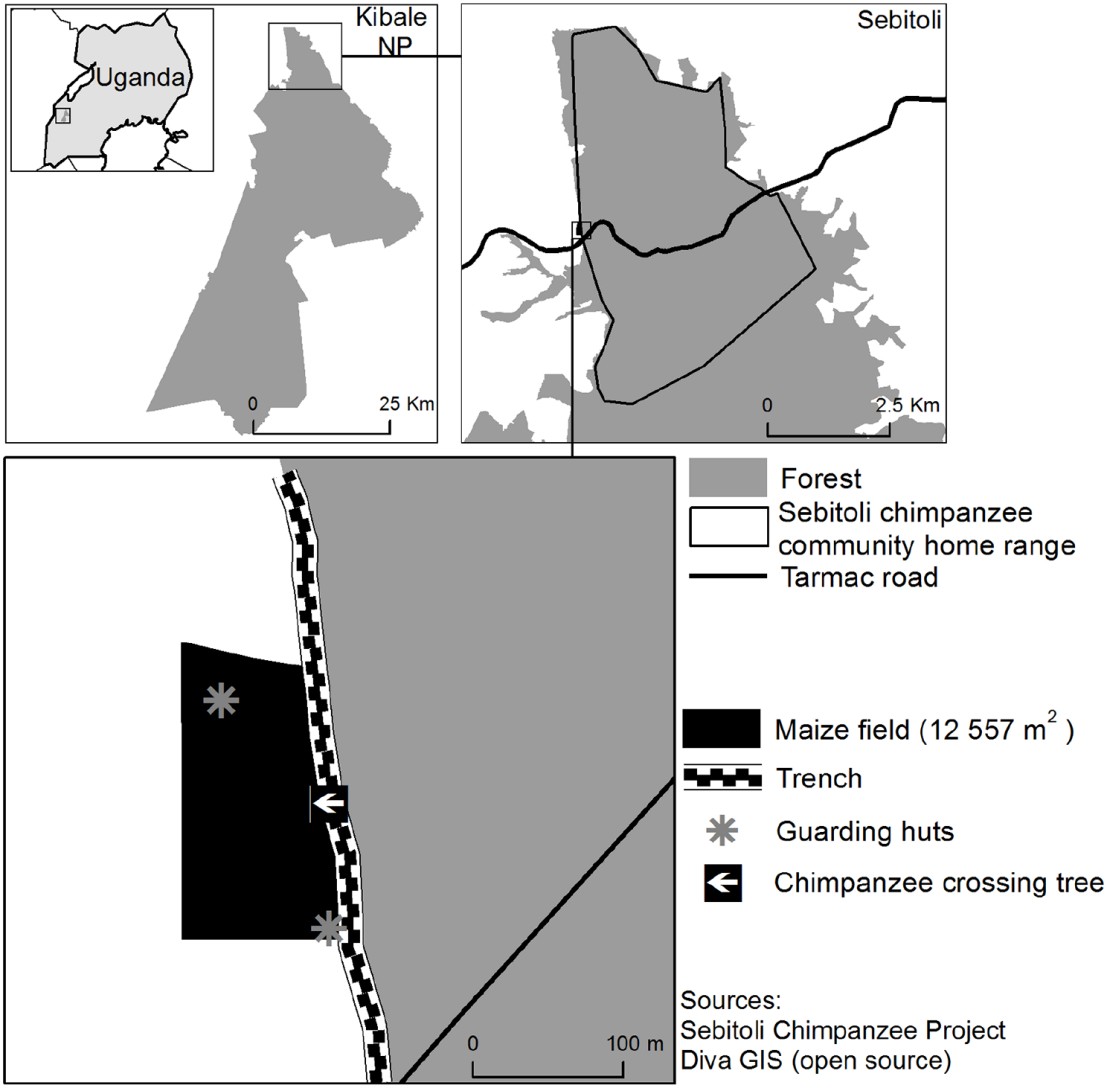
Uganda-Kibale National Park-Sebitoli area, home-range and maize field monitored (location of the guarding huts, the fallen tree and the video-trap).

During our routine chimpanzee monitoring in the North-Western part of the territory of Sebitoli chimpanzees (Figure 1), we regularly found maize remains (corn and stems) associated with chimpanzee footprints and faeces along a 2 meters wide per 2 meters deep trench, that was dug by the Uganda Wildlife Authority to prevent elephant from incurring and destroying the crops. At the time of the study, the efforts by the local communities to protect such crop fields were fairly significant: day and night guarding of the field by sleeping in a hut situated in the maize field (surface = 17,838 m^2^ calculated from GPS records; Garmin 450) at 35 meters from the trench where chimpanzee food remains were found and maintenance of the trench. However, in a location along the maize field, the two sides of the trench were bridged horizontally by a single fallen trunk (*Acanthus pubescens* species, diameter at breast height = 0.11 m). The maize food remains were often found piled up in front of the trunk showing that this point was the main and preferred access. According to footprints and feeding remains, the alternative of this direct access was a 100-meter diversion to access the crop, even though it cannot be excluded that some individuals may access the field in other ways thus the observed party size may have been underestimated. We placed on this bridge a HD video-trap (Bushnell Trophy Cam HD Max) with a day/night autosensor (motion sensor reaching out to 60 feet or beyond and IR flash i.e. a LED night vision flash that sends a burst of Infrared Energy which is invisible to the human eye and an adjustable Passive Infra Red (PIR) motion detector, with no-glow black LEDs) and sound recordings. The settings were the following: high definition video of 1280×720 pixels, video length of 30 seconds, trigger interval of 1 second, and low PIR. The video-trap started recording digital pictures when motion is sensed at a distance up to 18 m. For the study period, night is defined as the period between sunset and sunrise i.e. from 19∶07 to 7∶01 during the study period and at this location [52]. To better categorise nocturnal activities, we also consider the period of twilight defined as the illumination produced by sunlight scattering in the upper atmosphere. Twilight occurs between dawn and sunrise and between sunset and dusk. While several twilights are defined according to the position of sun below the horizon, we use in this study the nautical twilight, which is considered as complete darkness (sun is less than 12° below the horizon). In the studied area, twilight is short (crepuscular period is briefest at the Equator) and always last less than one hour [53].

**Figure 2:**
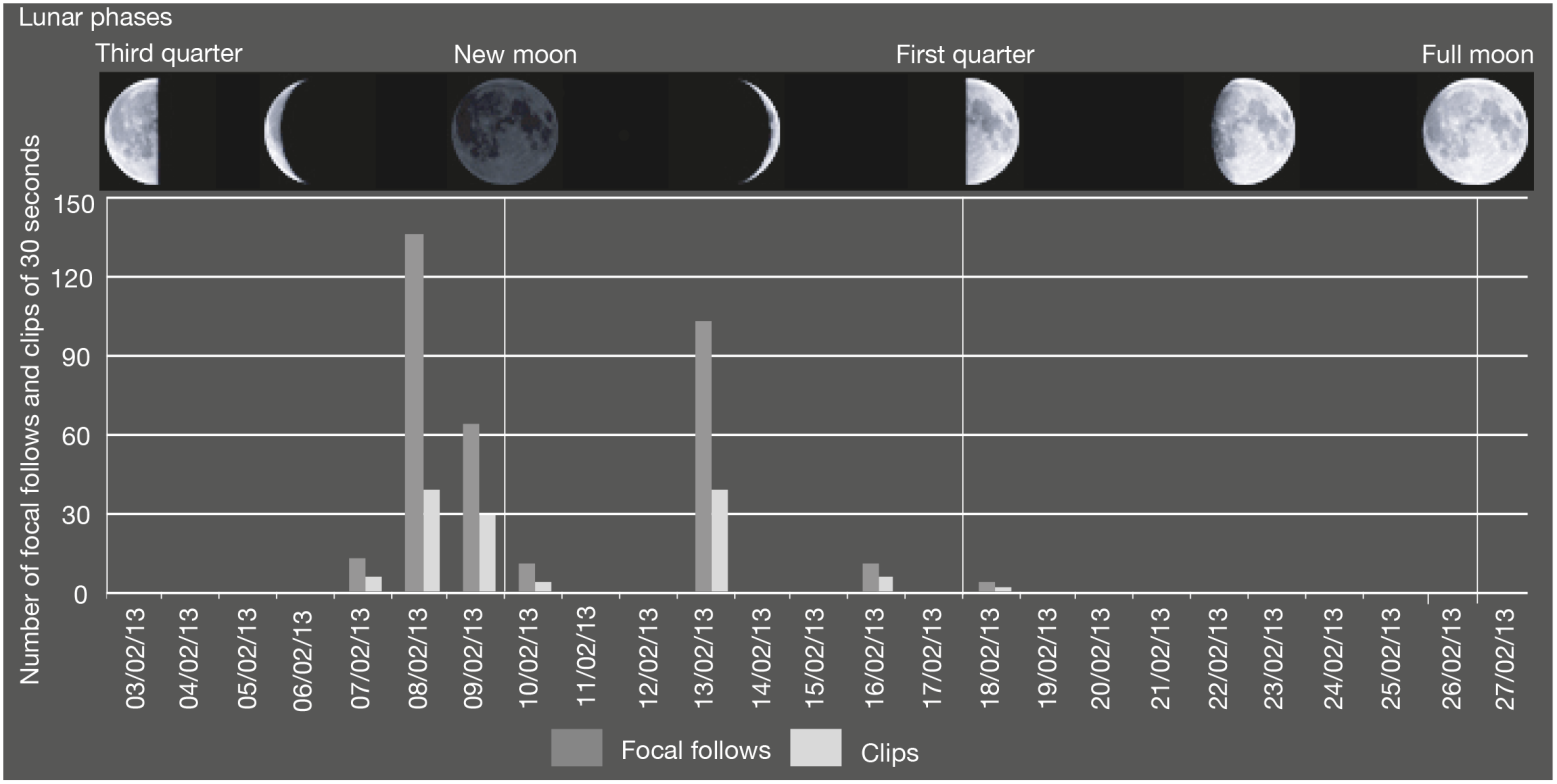
Number of clips and number of individual sessions recorded by the video-trap from the 5th to the 25th of February 2013 according to lunar phases during this period.

Infrared images are automatically recorded at night as well as during daytime if the light is low due to rainy or cloudy weather. The camera was fixed on a tree facing the small maize field on the forest side at 3.5 m from the trench. On recorded clips, we can clearly see the behaviour of the chimpanzees in the forest along the trench and on the fallen tree. They were not visible while inside the maize field, however as the maize is growing less than 2 m along the trench, the chimpanzees that crossed the fallen tree were entering the maize field. We identified the individuals visible in each 30 second-clip. However, we were not able to identify all individuals because recognition of chimpanzees that were not facing the video trap was difficult especially in the infra-red images. Finally we defined as “full moon” days, the days when the moon is completely illuminated, visible from sunset to sunrise and illuminance is about 0.3 to 1 lux. As opposite during “new moon” days, the moon is not visible or in his first visible crescent (illuminance is about 0.0001 lux). During first and third quarter lunar phases, 50% to 99% of the disc is visible and illuminance is about 0.01 lux (see Fig. 2) [54].

### 4. Video analysis

At the party level (hypothesis 1), a crop-raiding event started when the first chimpanzee was recorded entering the field after at least three hours without clip records and it ended when the last chimpanzee of the party (visible from the clip) was recorded leaving the field. At each event of crop-raiding, the party size (number of different individuals recorded by the clips), the party composition and the order of individuals crossing the bridging tree while entering and leaving the maize field were determined whenever possible. The composition of the entering party sometimes differed from the leaving one as individuals may enter or leave without being recorded on the video-clips.

At the individual level (hypothesis 2), a visit to the maize field started when the chimpanzee entered the maize field by coming down from the fallen trunk and ended when the individual climbed again onto the fallen tree to return towards the forest. However, not all the chimpanzees visible on the video-clips entered the maize field (at least using the fallen tree), with some of them staying at the forest edge, sitting or travelling along the trench or on the fallen trunk.

We recorded the behaviour of each visible chimpanzee (identified or not) in all clips. A clip in which n chimpanzees are visible produces n individual sessions lasting from 1′ to 30′. The set of clips n°1 to N°X will thus provide a total of N = n_1_+n_2_+….+n_X_ individual sessions. We counted the occurrence and the duration of each possible behaviour linked to anxiety, vigilance or to reduction of the risk of detection for each visible individual:

– gentle and rough self-scratching (as described in [55])
– scanning behaviours such as (i) guarding (standing in a quadrupedal posture for more than five seconds without moving [56]), (ii) bipedal position, (iii) arboreal scanning behaviours (i.e. climbing up in a tree bordering the targeted crop and watching in the direction of the field)
– defecation and its consistency: diarrhoea can be induced by fear and is used as an index of anxiety [57]
– vocalisations produced, associated behaviours and context (locomotion, feeding and social contexts)
– vigilance or waiting time at the edge of the forest before/after crossing the trench
– locomotion type (suspensory or quadrupedal arboreal locomotion used on the bridging tree) while the journey for crossing and its duration to either go into or come back from the field: the time each individual took to cross the trench from one end to the opposite side of the bridging tree.

### 5. Data analysis

The data set allowed only basic non-parametrical statistical approach because many observations did not enable reliable identification of individual chimpanzees. While the video-trap only captured the chimpanzees using the same access to the field and in some occasion do not record the full party, we compared the median of the party size during crop raiding and during feeding acitivities in the forest, this last one calculated under the chimpanzee habituation between February 3^rd^ 2009 and February 9^th^ 2013. It corresponds to the maximum number of chimpanzees observed during a feeding bout (N = 1 417; 61 577 minutes, mean = 43 minutes, SD = 65 min). A feeding bout was considered to start when at least a chimpanzee consumed a wild food item and ended when all the party members stopped eating. To compare the median of the feeding party size during crop raiding events and during usual feeding in the forest we used a Chi-Square test. To test for any sex/age differences in the diurnal or night time durations (i.e. time spent into the maize field) we used Mann Whitney U test since comparisons were made among different individuals: e.g. in the suitable data available – data on a whole crop-raiding visit of an identified individual – individuals who made the night visits to the maize field were not always the same individuals who made the day-time visits. For the same reason we used the same test to compare the speed of suspensory locomotion during night and day visits. To compare the occurrence of the behavioural signs of vigilance and anxiety between day and night incursions we used Wilcoxon exact test comparing the frequency of each behaviour per minute of video record in the two conditions (night and day).

## Results

### 1 Hypothesis 1: developing group specific behaviours to avoid detection in response to a risky situation

#### 1.1 Infrequent incursions into the maize field

During the 20 days of the study, a total of 14 crop-raiding events were recorded by the activation of the video-trap. Images of wild animals (chimpanzee, civet, red tail monkey) were captured in 122 clips of 30 seconds each during seven days and included 120 clips of chimpanzees for a total of 60 min of records. During the first part of the study period (February the 7^th^–the 10^th^), crop-raiding events occurred at least once a day (71 clips out of 120), and later, visits were spaced every one or two days (Figure 2).

#### 1.2 Crop-raiding in small parties

In the 14 journeys in which the number of chimpanzees entering or leaving the maize field was determined, the median of the party size was 8.20 chimpanzees (range: 3–17 individuals), thus more than double the median of the party size (3 chimpanzees; range: 1–30 individuals) of the same community during feeding activities in the forest (measured from 1 417 observations of 61 577 minutes).

#### 1.3 High ranking males leading the activities

**Figure 3:**
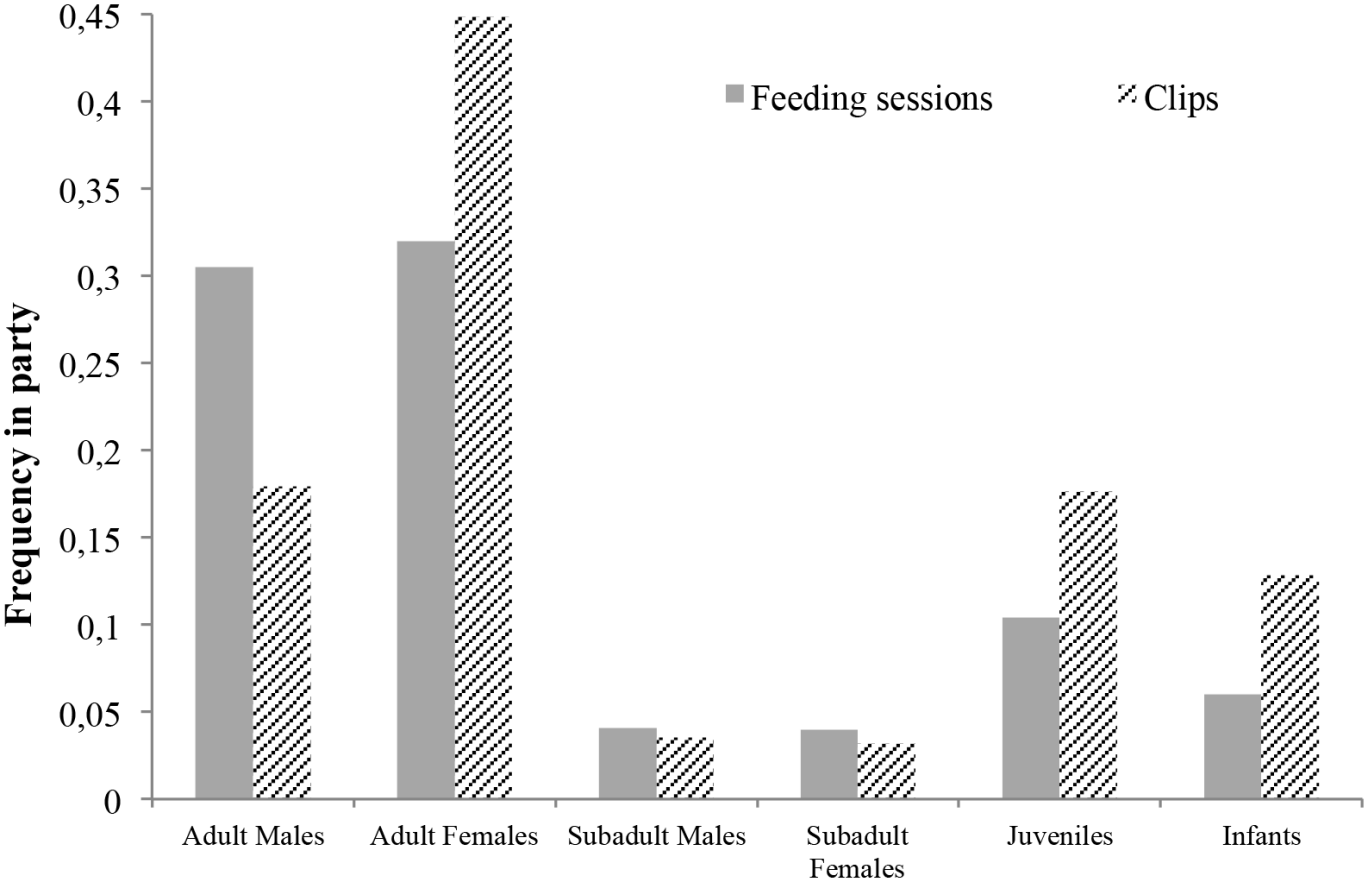
Party composition during forest feeding activities and activities at the border of the maize field.

The 120 clips of chimpanzee records provided 354 individual sessions. Those individual sessions in which sex and age class of individuals can be identified (330 out of 354) showed the presence of all age/sex classes (n of sessions for adult males: 56, adult females: 144, sub-adult females: 12, sub-adult males: 12, juveniles: 61, infants: 45) with a proportion not significantly different from the usual party composition of Sebitoli community during feeding bouts (Figure 3, Chi-Square = 0,232, df = 5, P = 0.999). In the 277 individual follows in which we were able to determine the exact identity of the individual (N_individual_ = 29; mean number of clips/individual = 9.60; range: 1–39), mean time in the videos/individual = 4′05″ (8′–25′28″), 139 observations correspond to females (55′47″), 138 to males (1h00’21″) and 134 correspond to chimpanzees going into the field, 119 coming back from the field, 24 being along the trench. Vulnerable individuals such as females with clinging infants (N = 3, respectively in 39, 5 and 2 clips) and severely mutilated individuals were recorded on clips, these include two individuals missing feet (1′24″ on 6 clips and 59″ in 3), a female missing four fingers to the left hand (3′03″ in 10 clips) and a juvenile with an unconsolidated broken leg (13′17″ in 33 clips). Mainly females led the party entering the maize field (five out of seven crop-raiding events).

### 2 Hypothesis 2: specific individual behaviours to limit the risk of being detected

#### 2.1 Being vigilant

**Figure 4:**
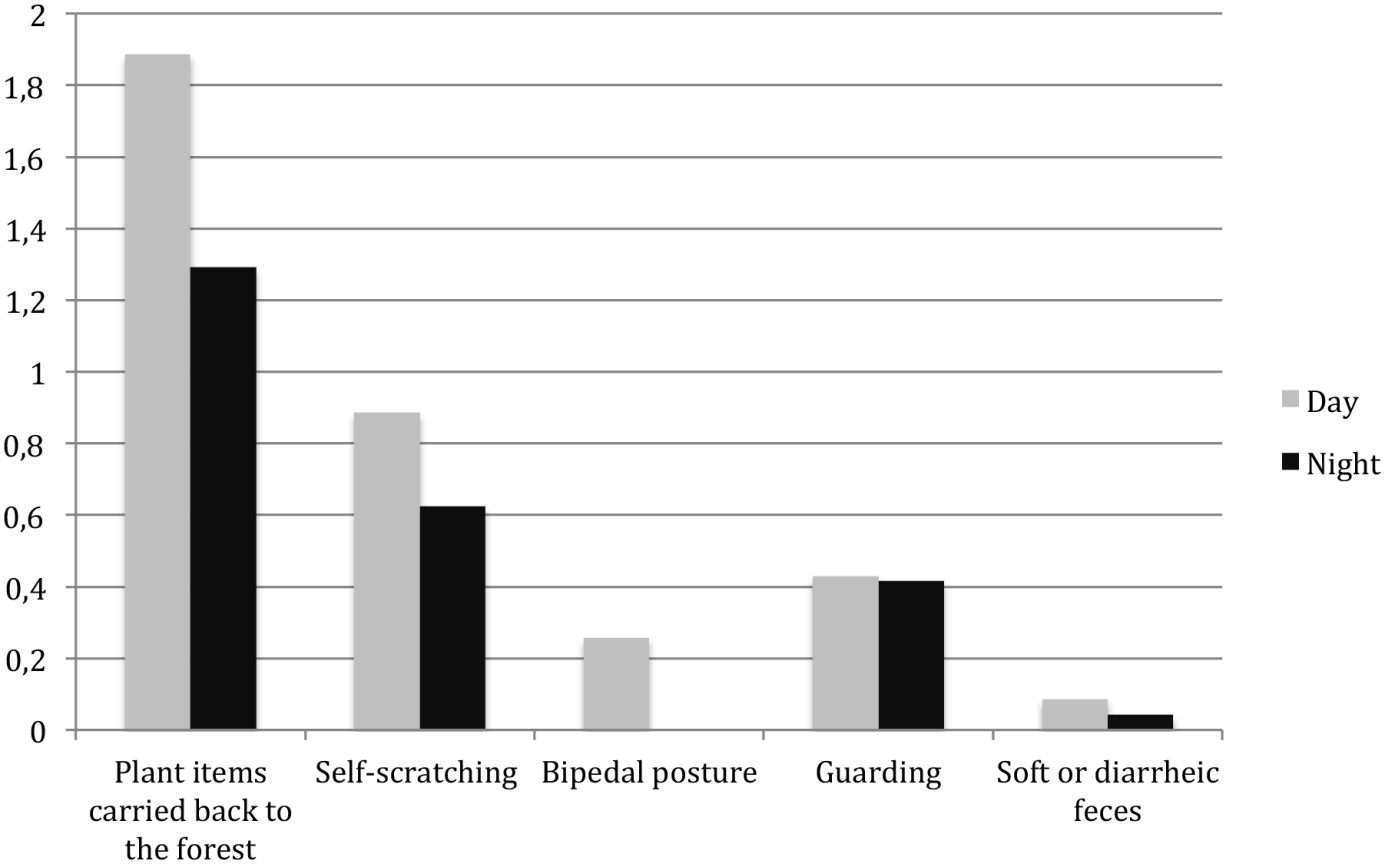
Frequency of signs of anxiety and vigilance in chimpanzees during day and night crop-raiding (occurrence of each behaviour per minute of video record).

Before entering the field, chimpanzees displayed scanning behaviours (25 guarding postures, seven bipedal postures, two arboreal scanning in a high eucalyptus tree growing at the border of the field; clip S1) (Figure 4) and some individuals were not entering the field, staying at the edge. While in the maize field, chimpanzees were sometimes chased by barking dogs (N = 3 clips) or run after by the guardian of the field (N = 1), who threw a branch towards a severely mutilated adult female who hurried to cross the bridging tree. The screams and barks of chimpanzees during these events and the records of self-scratching behaviour and emission of soft/diarrheic faeces (Figure 4) in other occasions also indicated anxiety and perception of a risky situation. In twenty-two cases, chimpanzees came back from the field with ears (one to six pieces) or stems (six pieces) (clip S2) and among the half of them who were identified, six were females (carrying 14 items), five were males (bringing back nine items). Chimpanzees rarely transport wild food. Such transport of food items may indicate that chimpanzees were not ease to consuming crops in the field and felt at risk by staying in the field.

#### 2.2 Being silent and focused on the risky activity

A total of 36 events of vocalisations were recorded during 34 clips: grunts were the most frequent type of vocalisations (5 foodgrunts and 23 social pantgrunts), and louder vocalizations such as whimpering (N = 4), high screams (N = 2), panthoots (N = 1) and waabark (N = 1) occurred at a lower frequency. All vocalisations, except the screams and bark, were produced in social or feeding contexts. Interestingly, other social behaviours, usually absent during tense or risky situations, have been observed at the border of the trench such as a copulation, a female-female genital inspection and genital touching (a sort of greeting also described in Bossou and Nimba’s chimpanzees [58]; clip S3).

#### 2.3 Rushing

In the six events of crop-raiding recorded, in which we can clearly identified the individuals entering and going out of the maize field, the raids ranged from a minimum duration of 7′24″ to a max of 2h29**′**02″. The individual mean duration of the visits to the maize field was 40′09″ (1′59″-2h26**′**39″) based on 25 visits of identified chimpanzees going into the field and coming back. Adult males do not spend a significantly longer time in the maize field in comparison to adult females (average duration: adult male = 1h25**′**11″ (3′56″-2h28**′**17″, N = 4), adult females = 20′51″ (1′59″-1h57**′**13″, N = 12) Mann Whitney U exact, U = 12, P = 0.226). Severely mutilated individuals (three males and one female) stayed on average 37′36″ (1′59″-2h25**′**16″) in the field (N = 7 visits). Both male and female chimpanzees visiting the field plantation were thus neither rushing to consume the maize nor leaving quickly the forest edging the maize field (average: females = 1′41″ (5″-12′54″), males = 1′57″ (4″-24′10″) from 21 tree crossings by 11 different females and 27 for 16 different males (Mann Whitney U Test, U = 281, P = 0.958).

#### 2.4 Extension of the period of activity to darkness

**Figure 5:**
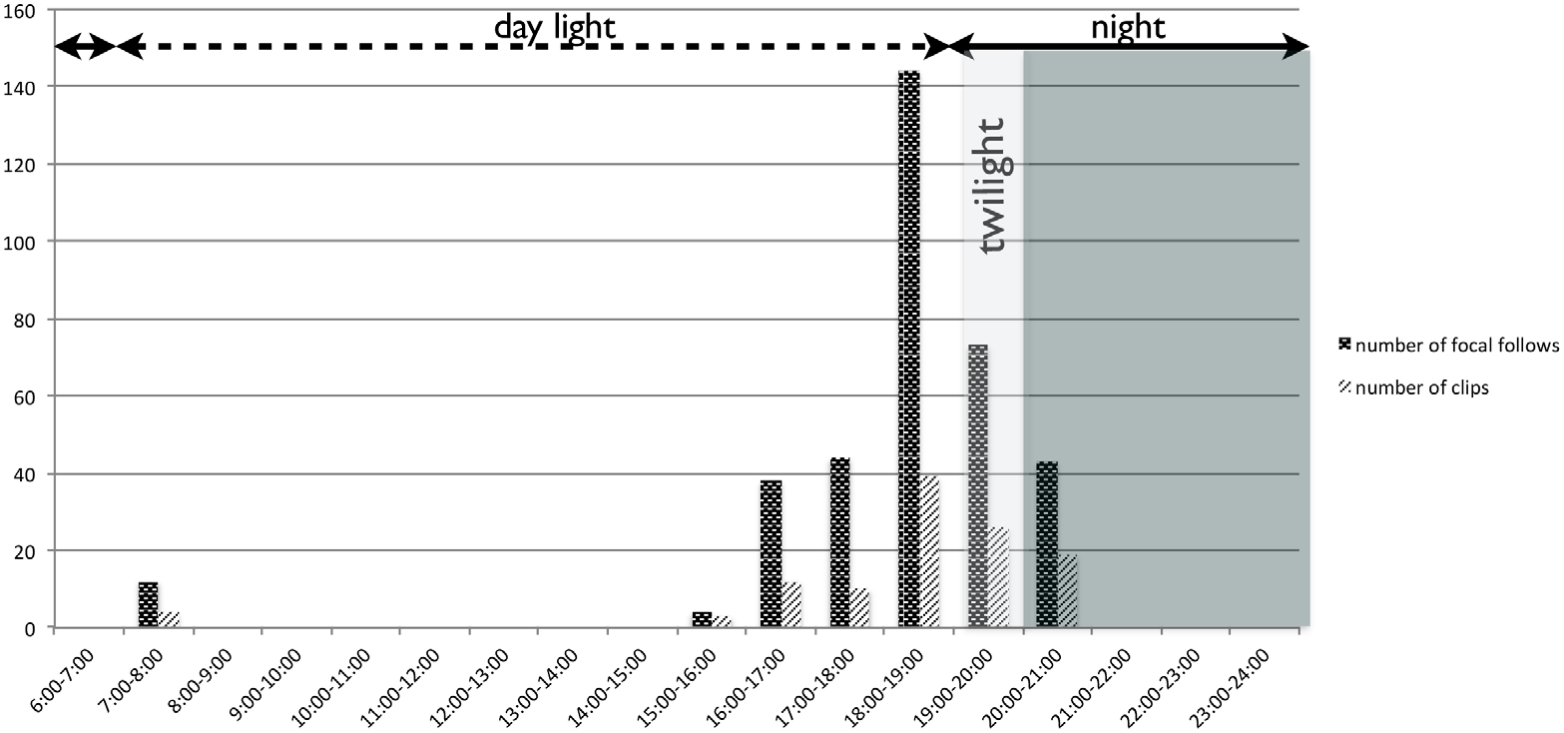
Time distribution of crop-raiding activities recorded (number of clips and individual sessions) during daylight and night including twilight and darkness period.

**Figure 6:**
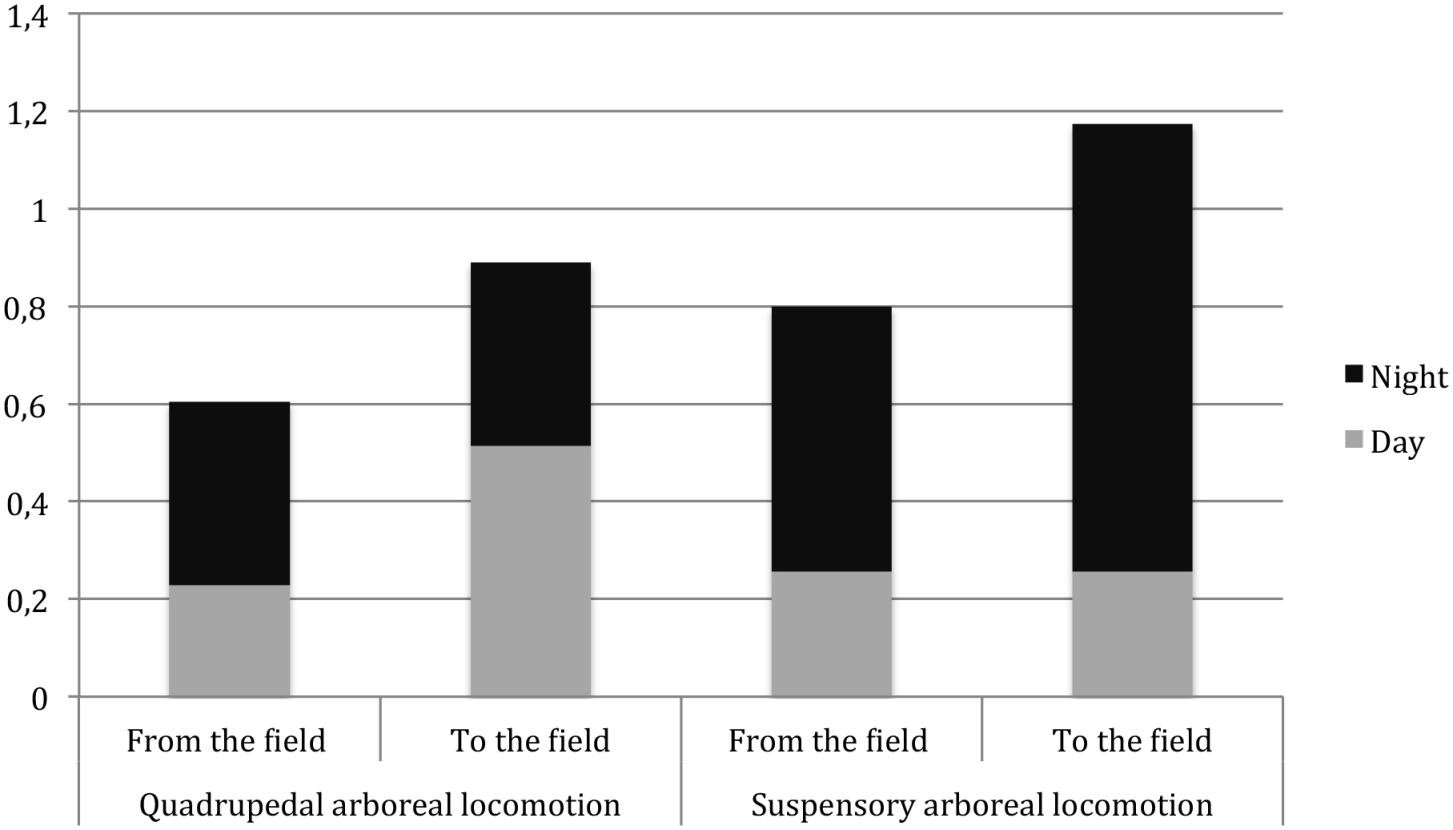
Types of arboreal locomotion used during night and day to cross the trench using the bridging tree to go and come back from the maize field (occurrence of each behaviour per minute of video record).

Forty one per cent of chimpanzee clips (N = 120) were recorded between sunfall (7∶07pm) and midnight, a time where chimpanzees are usually resting in arboreal night nests, 19 of them occurring after twilight. More than one third of nocturnal individual sessions (40 out of 114) occurred after twilight. Identification of chimpanzee at night in IR images was often challenging (10 night visits out of the 25 in which we can identify the individual both entering and coming out of the maize garden), however for seven instances the whole raid occurred for clearly identified chimpanzees after the sunset (range: 19∶21–19∶48). No clip was recorded before sunrise (7∶01 am), and only a short crop-raiding event (7′24″) by six individuals (four adult females and their dependants) occurred before 12.00 am. All night crop visits occurred during new moon, at the beginning of quarter moon or end of third quarter, thus when the night is darker than a full-moon period [53] (Figure 5). While during the day, chimpanzees generally stopped and sat on the bridging tree before entering the maize field, during night visits chimpanzees did not show hesitation and crossed rapidly the bridge to enter the field, using more frequently suspensory locomotion (66% vs 41% during daily visits), as opposed to quadrupedal locomotion during the day (Figure 6). In all nocturnal visits, even when chimpanzees screamed due to the noise of barking dogs, they did not flee from the maize field; instead they came back into the forest relatively slowly (clips S4, S5, S6). Adults and sub-adults spent a longer time in the maize field during night visits than during day light visits (median of the duration in the field: night = 1.120 (N = 7), day = 0.208 (N = 10); Mann Whitney U Test, U = 12, P = 0.024). Overall signs of vigilance and anxiety occurred all at higher frequency during the day than during night incursions (Figure 3; Wilcoxon exact test, T+ = 0, N = 5, P = 0.043).

## Discussion

Contrary to our expectations, despite the fact that crop-raiding have various costs perceived by the chimpanzees, the Sebitoli chimpanzees did not use behavioural strategies usually used in response to a risky situation. Instead, they display an innovative behaviour, raiding cultivated maize after sunset.

### 1. Repeated nocturnal activities outside of moonlit nights

The most surprising innovative behaviour captured on video traps is undoubtedly the night incursions into the maize field by Sebitoli chimpanzees. Indeed, chimpanzees preferred to visit the maize field during late afternoon or after sunset, staying twice as long in the field at night than during daylight. Some of the raids started before the sunset and continued after it but several of them occurred at twilight or even after it, in complete darkness. Nineteen clips out of 120 were recorded more than one hour after sunset, i.e. after twilight, when the illuminance is very low (0.0001–0,1 lux according to lunar phases during the study period [37], [38]. No other previous studies reported crop raiding after sunset. In Bossou, where chimpanzees consume 17 different types of cultivated foods, no relationship with time of the day has been recorded [45], [59] except for cassava (*Manihot esculenta*). The majority of cassava raids occurred during afternoon-time, with a greater-than-expected frequency [45], [59]. This suggests that cultivated sugar fruits attract chimpanzees who raid any time during the day, but for cassava, the high frequency at the end of the day suggests they probably first tried to feed on wild food but because of food scarcity they were pushed by hunger to take risks to obtain crops. In Sebitoli, the screams and barks of chimpanzees during crop-raiding, self-scratching behaviour and emission of soft/diarrheic faeces indicated anxiety and perception of a risky situation. Signs of anxiety and vigilance were less frequent after sunset than during day hours where they seemed tenser. At night, they also enter quickly into the field using suspensory locomotion showing less hesitation and do not rush when leaving it.

In fact, chimpanzee nocturnal raids were not marginal during our study period as 41% of the time spent at the border of the maize field (N = 120 clips i.e. 60 min) occurred during night hours. To our knowledge, this is the first report of long, repeated and group night activities by a great ape species outside of moonlit nights. As of today, the nightlife of chimpanzees has been neglected and we have probably missed some interesting activities as night-time lasts around half of each 24 h in the equatorial regions.

### 2. Chimpanzees raid in large parties including vulnerable members

Considering the context of Sebitoli where people are actively guarding their field at crop maturity, perception of local people who rarely report chimpanzee incursion and previous surveys conducted in the area of KNP, the number of 14 crop-raiding events in a short period of 20 days and the size and the composition of the parties participating are surprising. However the study period is to short to conclude about the frequency of crop-raiding in Sebitoli and to compare it to other sites like Bossou where crop raiding bouts range from 5 to 45 per month [34] but the absence of hesitation to enter the field at night as opposed to a day-light raid in the same area and behaviour in the neighbouring community of Kanyawara (Krief pers. observ.) indicates this is probably usual. During nocturnal activities, chimpanzees most likely feel more confident in coming out of forested areas to reach open fields to access highly rewarding food. Contrary to composition recorded in similar or other risky activities such as territorial patrols, intergroup conflicts and crop-raiding at other sites all biased towards males, in the Sebitoli incursions, all age/sex classes were involved, including females with clinging infants and severely mutilated individuals [16], [34], [45], [60]. Even though we acknowledge a small sample size, our data does not show evidence of specific leading behaviour of the alpha male or high ranking males to protect the individuals of the party entering into cultivated land. Adult females were even observed leading the incursion into the cultivation and the journey back to the forest, while party composition does not show a sex-ratio biased towards females.

During crop-raiding, we observed large party size (and this number is probably underestimated since the bridging tree may have not been the only way, even though preferential, to access the maize field), even larger than the habitual party size of the study community during feeding time. While a large party is more easily detectable during day, the cost of vigilance for each individual is likely reduced when travelling with more conspecifics, and during night when the visibility is reduced, numeric inconvenience is likely reduced. The fact that chimpanzees carry less maize from the field to the forest during night means they most likely consume it directly in the field. Indeed, emission of soft faeces, rough self-scratching, bipedal positions showed that the border zone slightly affected chimpanzee behaviour, which was anxious and vigilant, especially during day. However, they were not avoiding the location, even when humans were present, and did not really fear villagers that were guarding their crops, entering when present and staying close to the border after being chased. Previous surveys in the same park reported that chimpanzees rarely raid crops unless wild food availability is low [6]. Wilson et al. [29] considered such observations as evidence that chimpanzees are sensitive to the cost of crop-raiding and avoid the risk of being killed by humans. In contrast with patrols where chimpanzees are usually silent and as previously observed during crop-raiding for monkeys [29], [61], Sebitoli chimpanzees were not silent and did socially interact at the border of the field. However, loud calls were rare and the only male to pant-hoot in the maize field was the higher ranking male as observed previously during periphery excursions [29]. Actually, the production of loud calls recorded during excursions (also called “border checking” to distinguish them from patrols) was interpreted as advertising their presence and coalitionary strength to intimidate neighbours [16], [62].

### 3. Rapid adaptation to low predation risk and food with high nutritive value

Although few examples have been identified in primates, the ability to operate both at night and during the day is common among mammals [63]. Cathemeral animals are characterized by an extreme behavioural flexibility, and they may range from almost full diurnality to almost full nocturnality [63]. Our closest relative, the chimpanzee, does not show the physical adaptation to nocturnal activities found in other primate species, such as prosimians (e.g. large orbits and presence of *tapetum lucidum*, the reflective layer behind the retina to enhance available light). It is likely that in a diurnal species only multiple advantages would lead it to engage in the risky behaviour of cathemerality. Among the motivational causes suggested for cathemeral animals, three main ecological benefits may be the ultimate reasons for the activity shifts in chimpanzees: predator avoidance, the need to complement their dietary requirements and heat stress avoidance [39], [40], [41], [64], [65].

With regard to the first hypothesis, just a few predators (e.g. snakes, humans) might threaten Sebitoli chimpanzees given that leopards, which are the main nocturnal predator for this species, are so rare in Kibale NP that they could not represent a threat for chimpanzees ([36], [66], Chapman pers. comm.). Local human population, Bakiga and Batooro, do not eat chimpanzee meat but they protect their field with snares and in Sebitoli, chimpanzees are particularly affected since approximately 40% of the individuals are victims of severe mutilations (Sebitoli Chimpanzee Project, long term data). Field owners actively guard their cultures, sometimes resulting in serious injuries to wildlife intruders. The alpha-male of the Kanyawara chimpanzee community, neighbouring the Sebitoli community, has been severely injured by a spear [29], [30]. Chimpanzees may have adapted ancestral antipredatory and patrol behaviour to face new threats (i.e. human defence of crop fields, highway crossing) by using shift systems in their activity period and improving cooperative behaviours to maximise the protection of the party. However, while road crossing or boundary patrols usually occur during the day, the nocturnal crop-raiding activity displayed by Sebitoli chimpanzees emphasizes their high level of flexibility and capacity for rapid adaptation due to the likely predation by leopards 100 years ago in Kibale NP making such nocturnal visits risky at that time.

Secondly, such adaptation may provide a significant supplement to their natural diet adding food of higher nutritional quality (one ear of maize provides 86 Kcal and is particularly high in carbohydrate (19.2% compared to the average of 13.9% of 32 wild fruits from Kibale; [67], [68]. The consumption of several ears of maize represents a high intake, as daily energy intake for Kibale’s chimpanzees range from 1206 and 3333 Kcal on estimation [69].

Moreover, as usually in cathemeral species such flexibility may depend also on seasonal variations in food availability in the forest and/or seasonality in field cultivation for humans [41], [70], [71], [72], [73]. Therefore, the fact that the maize was mature when the wild food availability was not high (Intermediate Food Availability between January and February 2013 calculated after [46]), may have led hungry chimpanzees to risk more and take the opportunity of consuming highly nutritive food while caloric intake was not high during the day.

Interestingly the nocturnal behaviour in Sebitoli chimpanzees during new moon, first or third quarter (absence of moonlight) was inconsistent with previous records of cathemeral and nocturnal primates showing general lunarphilia (cathemeral primates [41], [74], nocturnal primates [75], [76], even after other rare recordings of night activities in other chimpanzee communities [16], [43]. Two alternative hypotheses may be formulated to explain such peculiar observations. The Sebitoli chimpanzees perform night crop-raiding during the period of the absence of moon light in order to be less detectable from humans or simply due to the maturation stage of the maize that may disappear if they waited for the full moon.

However, in chimpanzees, the absence of physical and physiological adaptations to nocturnal vision raises the question of how they are able to raid crops during dark nights. The high anthropogenic pressure in the Sebitoli area deeply altered the canopy and the forest cover. During the 60′s and 70′s, half of the trees were destroyed by logging activities in almost the whole home-range of Sebitoli chimpanzees [48]. Therefore, chimpanzees live today in a more open habitat with higher light penetration at night than before and than in other more pristine forests. Such increased light penetration may encourage them to travel and forage later after sunset in crop plantations at the periphery of the Park and to extend their activity over the 12-h gaining access to higher energy and quality food. Such behaviour is observed in *Eulemur fulvus*, mostly diurnal during long days of austral summer, which becomes as active at night during austral winter [41].

Indeed, as for other animal species, cathemerality seems to be linked to a particular habitat, thus the exceptional nocturnal activity of chimpanzees may be related to the peculiar feature of the study area, a region where habitat loss and human population expansion have resulted in severe encroachment on wildlife habitat [77], [78]. While in South-Western Uganda early agriculture through forest clearing and environmental degradation started 4800 years ago [79], today the quasi totality of Ugandan forested areas (98%) are considered as regenerated forests, and the remaining 2% are planted forest exclusively with introduced species [80] (e.g. eucalyptus, pines, cypresses [81]). This situation contrasts with the world’s forests where primary forests (forest of native species that have no clearly visible indications of recent human activities) account for 36% and regenerated forests for 57% of the total forest cover [80]. Additionally, considering the non negligible amount of crops consumed by chimpanzees, it is urgent to reconsider fertilizers and pesticides used in crops to avoid contamination of wild surrounding habitat, soil and sediments and to promote organic agriculture at the interface with wildlife. The high frequency of congenital deformities observed in the Sebitoli chimpanzee community of this study may be related to the exposure to chemicals while crop raiding [47].

### 4. Conclusions

Even though the chimpanzees’ home range has been seriously damaged and disturbed by both logging activities and significant human demographic pressure, chimpanzees have shown great behavioural flexibility including unexpected nocturnal behaviour, in order to take advantage of the proximity of domestic nutritive food. The new findings of chimpanzee nocturnal raids can aid to formulate recommendations to local farmers and Park authorities in addition to those already listed as “best practice guidelines” from IUCN in terms of human-wildlife conflicts [82].

## Supporting Information

Clip S1 **13/02/2013, 18h01′00**″ **(duration 30″) (daylight).** **In this clip, an individual (not identified) climbs the eucalyptus tree at the edge of the maize field for arboreal scanning.** An adult male and adult swelling female cross the tree, both in quadrupedal arboreal locomotion, followed by another adult male who crosses in suspension. Lastly, SA, a sub-adult swelling female arrives and stands at the border of the trench.
(AVI)

Clip S2 **13/02/2013, 18h05′09**″ **(duration 30″) (daylight).** This clip shows an adult male (NE) who comes back from the maize field with six ears of maize (held by the mouth, by the right hand and between the left arm and his body), a juvenile male (KI) sitting on the bridging tree, his mother (KU) and another adult female (KL) with clinging infant (KR) approaching the bridging tree from the forest side.
(AVI)

Clip S3 **13/02/2013, 18h05′45**″ **(duration 30″) (daylight).** In this clip the old adult female (KU) touches the genital area of a female (KL) with clinging infant (KR), a behaviour described as a sort of greeting [56]. Then KL (with KR carried ventrally) crosses the bridge in suspension, while KU together with another adult female climbed the bridging tree where an adult male (NE) and his juvenile son (KI) are sitting. A juvenile male (UL) faces the camera trap.
(AVI)

Clip S4 **13/02/2013–20h15′27**″ **(duration 30″) (night time, IR image).** A chimpanzee coming from the field (with a maize ear in his mouth) crosses the bridge in quadrupedal arboreal locomotion and then in suspension to pass around another individual who is feeding maize (stem and ear) on the bridging trunk.
(AVI)

Clip S5 **13/02/2013–20h24′47**″ **& 20h25′23**″ **(duration 2×30″) (night time, IR image).** In these clips, some screams and waa-barks are emitted, then a mutilated adult chimpanzee (BM, whose left foot is missing) is coming back from the maize field followed by four individuals (including one holding a maize ear with the mouth). Among them ET, the alpha-male is pilo-erected when crossing in suspension, but then sits across the trench, facing the maize field.
(AVI)

Clip S6 **13/02/2013–20h24′47**″ **& 20h25′23**″ **(duration 2×30″) (night time, IR image).** In these clips, some screams and waa-barks are emitted, then a mutilated adult chimpanzee (BM, whose left foot is missing) is coming back from the maize field followed by four individuals (including one holding a maize ear with the mouth). Among them ET, the alpha-male is pilo-erected when crossing in suspension, but then sits across the trench, facing the maize field.
(AVI)
